# Supplementary material for: Understanding women’s care experiences in public health facilities in a conflict-affected area of Nigeria
Source: PLOS Glob Public Health. 2025 Dec 23;5(12):e0004563. doi: 10.1371/journal.pgph.0004563 (PMC12725616; doi:10.1371/journal.pgph.0004563)
Supplement: S1 File — (DOCX) [file pgph.0004563.s001.docx]

| **FACILITY TYPE** | |
| --- | --- |
| Health facility visited (name): |  |
| Type of health facility | Tertiary/referral hospital [1]  Secondary/general hospital [2]  Comprehensive health center [3]  Primary health center [4]  Basic health center/health post [5]  Other (specify)………………………………………..[95]  __________________________________________ |
| Managing authority | Government [1]  Mission/faith based [2]  NGO / Private not-for-profit [3]  Private for profit [4]  Other (specify)………………………………………..[95]  __________________________________________ |
| **FACILITY LOCATION** | |
| Province/State Name: |  |
| Health Zone/LGA Name: |  |
| City / Village Name: |  |
|  |  |
| **DATA COLLECTION TEAM** | |
| Name of Data Collector: |  |
| Name of Team Lead: |  |
| Date of Visit: (dd/mm/yy) | __ __ / __ __ / __ __ |
| Time of Visit: (hh:mm) | __ __ :__ __ |

**Supplementary File 1: Exit Survey with women**

| SECTION 1: INFORMATION ABOUT BIRTH | | | | | | | |
| --- | --- | --- | --- | --- | --- | --- | --- |
| **101**: Did you plan to give birth at this facility? | **CODE** |  | |  | |  | |
| Yes | 1 |  | |  | | Go to 104 | |
| No, I had a problem during delivery at home | 2 |  | |  | | Go to 103 | |
| No, other reason | 3 |  | |  | | Go to 102 | |
| **102**: Did you plan to give birth at a different facility? | 1 | 0 | |  | |  | |
| **103**: What was the main reason you did not plan to deliver at a facility?  *IF CLIENT MENTIONS SEVERAL REASONS, PROBLE FOR THE MOST IMPORTANT OR MAIN REASON. IF MAIN REASON IS NOT LISTED, SELECT ‘OTHER’ AND INCLUDE REASON GIVEN* | **CODE** |  | |  | |  | |
| Inconvenient location | 1 |  | |  | |  | |
| Delivering at a facility is unnecessary | 2 |  | |  | |  | |
| Bad previous experience at health facilities | 3 |  | |  | |  | |
| Afraid of being cut | 4 |  | |  | |  | |
| Lack of privacy at facilities | 5 |  | |  | |  | |
| Cost | 6 |  | |  | |  | |
| Lack of supportive staff at facility | 7 |  | |  | |  | |
| Other (specify) | 95 | ______________________________ | | | | | |
| Don’t know | 98 |  | |  | |  | |
| **104**: What month and day was your baby born? | Month (mm) ___________  Day (dd)_____________ | | | | | | |
| **105:** Was your baby born via natural (vaginal) or cesarean birth? | **CODE** | | | | | | |
| Vaginal birth | 1 | | | | | | |
| Cesarean section | 2 | | | | | | |
| **106**: What time of day was your baby born*?* | *USE 24 HOUR CLOCK*   \|  \|  \|  \|  \| \| --- \| --- \| --- \| --- \| | | | | | | |
| **107**: Is your baby a boy or a girl? | **CODE** | | | | | | |
| Boy | 1 | | | | | | |
| Girl | 2 | | | | | | |
| **Question** | **Yes** | | **No** | | **DK/NA** | | **Go to** |
| Now I am going to ask you some questions about how you were treated during your time at this facility.  **108**: During labor, did you want to have someone outside of facility staff, such as a family member or friend, in the room to support you? | 1 | | 0 | |  | | No→109 |
| **108a**: Were you able to have that person with you during labor? | 1 | | 0 | |  | |  |
| **109**: During childbirth, did you want to have someone outside of facility staff, such as a family member or friend, in the room to support you? | 1 | | 0 | |  | | No→110 |
| **109a**: Were you able to have that person with you during childbirth? | 1 | | 0 | |  | |  |

| Question | Yes | No | DK/NA | Go to | |
| --- | --- | --- | --- | --- | --- |
| **110**: After birth, did you want to have someone outside of facility staff, such as a family member or friend, in the room to support you? | 1 | 0 |  | No→111 | |
| **110a**: Were you able to have that person with you after birth? | 1 | 0 |  |  | |
| **111**: After your baby was born, were you and your baby separated for more than one hour at any one time? | 1 | 0 | 98 | |  |
| **112**: After your baby was born, did your baby receive any other liquids or foods other than breast milk? | 1 | 0 | 98 | |  |
| **113**: Before you were discharged from the facility for this delivery, did any health care worker talk to you about taking care of yourself and your baby after delivery? | 1 | 0 | 98 | | No/DK→114 |
| **114**: Which topics did the health worker tell you about?  *READ EACH TOPIC AND RECORD THE CLIENT’S ANSWER* |  |  |  | |  |
| **114a**: Using family planning after the birth of your baby to prevent unwanted pregnancy or space your next birth | 1 | 0 |  | |  |
| **114b**: Exclusive breastfeeding (not giving your baby any fluids or food in addition to breastmilk) | 1 | 0 |  | |  |
| **114c**: Where to access breastfeeding support in the community | 1 | 0 |  | |  |
| **114d**: Signs that the baby has had enough to eat | 1 | 0 |  | |  |
| **114e**: Signs that the baby is hungry | 1 | 0 |  | |  |
| **114f**: Risks of using feeding bottles, teats and pacifiers | 1 | 0 |  | |  |
| **114g**: Nutrition (what is good for you to be eating after having your baby) | 1 | 0 |  | |  |
| **114h**: The importance of taking iron folic acid tablets after having your baby | 1 | 0 |  | |  |
| **114i**: What to do if you feel sad or depressed after giving birth | 1 | 0 |  | |  |
| **114j**: Health signs and symptoms for which you must immediately come back to the facility | 1 | 0 |  | |  |
| **114k**: Health signs and symptoms for which you must immediately bring your baby back to the facility | 1 | 0 |  | |  |
| **114l**: Registration of the birth of your baby | 1 | 0 |  | |  |
| **114m**: Vaccinating your baby | 1 | 0 |  | |  |
| **114n**: How to engage and play with your baby | 1 | 0 |  | |  |
| **114o**: When to next visit a health facility for health checks for you and the baby | 1 | 0 |  | |  |
| ***END OF SECTION 1*** | | | | | |

| SECTION 2: EXPERIENCE OF CARE DURING LABOR AND DELIVERY | | | | |
| --- | --- | --- | --- | --- |
| Thank you for answering my questions about your delivery. Now I am going to ask you some more questions about specific aspects of your delivery experience. I know some of these are difficult to remember, so it is ok if you don't, but try to tell me what you can as it will be useful in understand the quality of care in this facility. | | | | |
| **201**: Did the doctors, midwives or other healthcare providers call you by your name? *IF YES, PROBE:* Would you say this was all the time, most of the time, or a few times? | **CODE** |  |  |  |
| No, never | 0 |  |  |  |
| Yes, a few times | 1 |  |  |  |
| Yes, most of the time | 2 |  |  |  |
| Yes, all of the time | 3 |  |  |  |
| Don’t know / can’t remember | 98 |  |  |  |
| **202**: Did the doctors, midwives or other healthcare providers treat you with respect? *IF YES, PROBE:* Would you say this was all the time, most of the time, or a few times? | **CODE** |  |  |  |
| No, never | 0 |  |  |  |
| Yes, a few times | 1 |  |  |  |
| Yes, most of the time | 2 |  |  |  |
| Yes, all of the time | 3 |  |  |  |
| Don’t know / can’t remember | 98 |  |  |  |
| **203**: Did the doctors, midwives or other healthcare providers treat you in a friendly manner? *IF YES, PROBE:* Would you say this was all the time, most of the time, or a few times? | **CODE** |  |  |  |
| No, never | 0 |  |  |  |
| Yes, a few times | 1 |  |  |  |
| Yes, most of the time | 2 |  |  |  |
| Yes, all of the time | 3 |  |  |  |
| Don’t know / can’t remember | 98 |  |  |  |
| **204**: During examinations in the labor room, were you covered up with a cloth or blanket or screened with a curtain so that you did not feel exposed? *IF YES, PROBE:* Would you say this was all the time, most of the time, or a few times? | **CODE** |  |  |  |
| No, never | 0 |  |  |  |
| Yes, a few times | 1 |  |  |  |
| Yes, most of the time | 2 |  |  |  |
| Yes, all of the time | 3 |  |  |  |
| Don’t know / can’t remember | 98 |  |  |  |
| **205**: Did the feel like the health workers involved you in decisions about your care? *IF YES, PROBE:* Would you say this was all the time, most of the time, or a few times? | **CODE** |  |  |  |
| No, never | 0 |  |  |  |
| Yes, a few times | 1 |  |  |  |
| Yes, most of the time | 2 |  |  |  |
| Yes, all of the time | 3 |  |  |  |
| Don’t know / can’t remember | 98 |  |  |  |
| Not applicable / no decisions | 99 |  |  |  |

| 206: Did the health workers ask your permission before doing examinations and procedures on you? *IF YES, PROBE:* Would you say this was all the time, most of the time, or a few times? | CODE |  |  |  |
| --- | --- | --- | --- | --- |
| No, never | 0 |  |  |  |
| Yes, a few times | 1 |  |  |  |
| Yes, most of the time | 2 |  |  |  |
| Yes, all of the time | 3 |  |  |  |
| Don’t know / can’t remember | 98 |  |  |  |
| **207**: During the delivery, did you feel like you were able to be in the position of your choice? *IF YES, PROBE:* Would you say this was all the time, most of the time, or a few times? | **CODE** |  |  |  |
| No, never | 0 |  |  |  |
| Yes, a few times | 1 |  |  |  |
| Yes, most of the time | 2 |  |  |  |
| Yes, all of the time | 3 |  |  |  |
| Don’t know / can’t remember / not applicable (CS) | 98 |  |  |  |
| **208**: Did the health workers explain to you why there were carrying our exams or procedures? *IF YES, PROBE:* Would you say this was all the time, most of the time, or a few times? | **CODE** |  |  |  |
| No, never | 0 |  |  |  |
| Yes, a few times | 1 |  |  |  |
| Yes, most of the time | 2 |  |  |  |
| Yes, all of the time | 3 |  |  |  |
| Don’t know / can’t remember | 98 |  |  |  |
| **209**: Did the health workers explain to you why and how they were giving you any medicines? *IF YES, PROBE:* Would you say this was all the time, most of the time, or a few times? | **CODE** |  |  |  |
| No, never | 0 |  |  |  |
| Yes, a few times | 1 |  |  |  |
| Yes, most of the time | 2 |  |  |  |
| Yes, all of the time | 3 |  |  |  |
| Don’t know / can’t remember | 98 |  |  |  |
| Not applicable / no medicines given | 99 |  |  |  |
| **210**: Did you feel like you could ask the health workers any questions you had? *IF YES, PROBE:* Would you say this was all the time, most of the time, or a few times? | **CODE** |  |  |  |
| No, never | 0 |  |  |  |
| Yes, a few times | 1 |  |  |  |
| Yes, most of the time | 2 |  |  |  |
| Yes, all of the time | 3 |  |  |  |
| Don’t know / can’t remember | 98 |  |  |  |
| 211: Did the health workers talk to you about how you were feeling? *IF YES, PROBE:* Would you say this was all the time, most of the time, or a few times? | **CODE** |  |  |  |
| No, never | 0 |  |  |  |
| Yes, a few times | 1 |  |  |  |
| Yes, most of the time | 2 |  |  |  |
| Yes, all of the time | 3 |  |  |  |
| Don’t know / can’t remember | 98 |  |  |  |
|  |  |  |  |  |
| **212**: When you needed help, did you feel the health workers paid attention? *IF YES, PROBE:* Would you say this was all the time, most of the time, or a few times? | **CODE** |  |  |  |
| No, never | 0 |  |  |  |
| Yes, a few times | 1 |  |  |  |
| Yes, most of the time | 2 |  |  |  |
| Yes, all of the time | 3 |  |  |  |
| Don’t know / can’t remember | 98 |  |  |  |
| **213**: Did you feel the health workers took the best care of you? *IF YES, PROBE:* Was this all the time, most times, or a few times? | **CODE** |  |  |  |
| No, never | 0 |  |  |  |
| Yes, a few times | 1 |  |  |  |
| Yes, most of the time | 2 |  |  |  |
| Yes, all of the time | 3 |  |  |  |
| Don’t know / can’t remember | 98 |  |  |  |
| **214**: Did you feel the health facility environment, including the washrooms, were clean? *IF YES, PROBE:* Would you say this was all the time, most of the time, or a few times? | **CODE** |  |  |  |
| No, never | 0 |  |  |  |
| Yes, a few times | 1 |  |  |  |
| Yes, most of the time | 2 |  |  |  |
| Yes, all of the time | 3 |  |  |  |
| Don’t know / can’t remember | 98 |  |  |  |
| **215**: Would you say you were treated differently because of any personal attribute, like age, marital status, number of children, language spoken, education, disability or other traits? *IF YES, PROBE:* Was this all the time, most times, or a few times? | **CODE** |  |  |  |
| No, never | 0 |  |  |  |
| Yes, a few times | 1 |  |  |  |
| Yes, most of the time | 2 |  |  |  |
| Yes, all of the time | 3 |  |  |  |
| Don’t know / can’t remember | 98 |  |  |  |
| **216**: Did you feel like you were treated roughly, for instance were you pushed, slapped, squeezed, pinched, physically restrained, or physically mistreated in any other way? *IF YES, PROBE:* Was this all the time, most of the time, or a few times? | **CODE** |  |  |  |
| No, never | 0 |  |  |  |
| Yes, a few times | 1 |  |  |  |
| Yes, most of the time | 2 |  |  |  |
| Yes, all of the time | 3 |  |  |  |
| Don’t know / can’t remember | 98 |  |  |  |
| **217**: Did you feel the health workers shouted at you, scolded you, insulted, threatened, talked to you rudely or verbally mistreated you in any other way? *IF YES, PROBE:* Would you say this was all the time, most of the time, or a few times? | **CODE** |  |  |  |
| No, never | 0 |  |  |  |
| Yes, a few times | 1 |  |  |  |
| Yes, most of the time | 2 |  |  |  |
| Yes, all of the time | 3 |  |  |  |
| Don’t know / can’t remember | 98 |  |  |  |
| ***END OF SECTION 2*** | | | | |
|  | | | | |
| **SECTION 3: ACCESS TO CARE** | | | | |
| Thank you for answering my questions about your delivery experience. Now I am going to ask you a few questions about common problems clients have at health facilities. As I mention each one, please tell me whether any of these were problems for you, and if so, whether they were major or minor problems for you. | | | | |
| **301**: Was the amount of time you waited to see the health worker a problem? *IF YES, PROBE:* Would you say this was a major problem or minor problem? | **CODE** |  |  |  |
| No, not a problem | 0 |  |  |  |
| Yes, a minor problem | 1 |  |  |  |
| Yes, a major problem | 2 |  |  |  |
| Don’t know / can’t remember | 98 |  |  |  |
| **302**: Were the hours of service at this facility (when the facility opens and closes) a problem? *IF YES, PROBE:* Would you say this was a major problem or minor problem? | **CODE** |  |  |  |
| No, not a problem | 0 |  |  |  |
| Yes, a minor problem | 1 |  |  |  |
| Yes, a major problem | 2 |  |  |  |
| **303**: Were the number of days services are available to you at this facility a problem? *IF YES, PROBE:* Would you say this was a major problem or minor problem? | **CODE** |  |  |  |
| No, not a problem | 0 |  |  |  |
| Yes, a minor problem | 1 |  |  |  |
| Yes, a major problem | 2 |  |  |  |
| **304**: Was the cost for services or treatments at this facility a problem? *IF YES, PROBE:* Would you say this was a major problem or minor problem? | **CODE** |  |  |  |
| No, not a problem | 0 |  |  |  |
| Yes, a minor problem | 1 |  |  |  |
| Yes, a major problem | 2 |  |  |  |
| **305**: Is this the closest facility to your home? | **CODE** |  |  | IF NO 🡪 306 |
| No | 0 |  |  |  |
| Yes | 1 |  |  |  |
| Don’t know | 98 |  |  |  |
| **306**: What was the main reason you did not go to the facility nearest to your home? *IF CLIENT MENTIONS SEVERAL REASONS, PROBE FOR THE MOST IMPORTANT OR MAIN REASON* | **CODE** |  |  |  |
| Inconvenient operating hours | 1 |  |  |  |
| Bad reputation | 2 |  |  |  |
| Don’t like personnel | 3 |  |  |  |
| No medicine | 4 |  |  |  |
| Prefers to remain anonymous (go where not known to people) | 5 |  |  |  |
| It is more expensive | 6 |  |  |  |
| Was referred | 7 |  |  |  |
| Service not offered at facility closest to home | 8 |  |  |  |
| Other, specify | 95 | _______________________ | | |
| Don’t know / can’t remember | 98 |  |  |  |
| ***END OF SECTION 3*** | | | | |

| SECTION 4: DELIVERY CLIENT PERSONAL CHARACTERISTICS | | | | |
| --- | --- | --- | --- | --- |
| **401**: How old are you? | Age in years | | | \|  \|  \| \| --- \| --- \| |
|  |  | | |  |
| **402**: Have you ever attended school? | **CODE** |  |  |  |
| No | 0 |  |  | If no 🡪 404 |
| Yes | 1 |  |  |  |
| Prefer not to say | 99 |  |  | If 99 🡪404 |
| **403**: What is the highest level of school you attended? | **CODE** |  |  |  |
| Primary school | 1 |  |  |  |
| Secondary school | 2 |  |  |  |
| Higher education | 3 |  |  |  |
| Prefer not to say | 99 |  |  |  |
| **404**: How many times have you given birth, before this delivery?  *PROBE: PLEASE INCLUDE STILLBIRTHS OR ANY CHILDREN WHO WERE BORN ALIVE AND LATER DIED* | Number of births | | | \|  \|  \| \| --- \| --- \| |
|  | This is the first birth | | | (ENTER 00) |
| **405**: Are you currently married or living together with a man as if married? | **CODE** |  |  |  |
| No, not in union | 0 |  |  |  |
| Yes, currently married | 1 |  |  |  |
| Yes, currently living with a man as if married | 2 |  |  |  |
| Prefer not to say | 99 |  |  |  |
| **406**: During the last year, were you or members of your household displaced from your home for a month or more? | **CODE** |  |  |  |
| No | 0 |  |  |  |
| Yes | 1 |  |  |  |
| Prefer not to say | 99 |  |  |  |
| Please indicate how much you agree with the following statements:  **407:** I feel safe from crime and violence when inside this health facility | **CODE** |  |  |  |
| Strongly agree | 1 |  |  |  |
| Agree | 2 |  |  |  |
| Disagree | 3 |  |  |  |
| Strongly disagree | 4 |  |  |  |
| Don’t know | 98 |  |  |  |
| Prefer not to say | 99 |  |  |  |
| **408:** I feel safe from crime and violence when traveling to and from this health facility | **CODE** |  |  |  |
| Strongly agree | 1 |  |  |  |
| Agree | 2 |  |  |  |
| Disagree | 3 |  |  |  |
| Strongly disagree | 4 |  |  |  |
| Don’t know | 98 |  |  |  |
| Prefer not to say | 99 |  |  |  |
| **409:** The area surrounding this health facility is safe and peaceful overall | **CODE** |  |  |  |
| Strongly agree | 1 |  |  |  |
| Agree | 2 |  |  |  |
| Disagree | 3 |  |  |  |
| Strongly disagree | 4 |  |  |  |
| Don’t know | 98 |  |  |  |
| Prefer not to say | 99 |  |  |  |
| **410:** The area surrounding this health facility is marked by repeated violence | **CODE** |  |  |  |
| Strongly agree | 1 |  |  |  |
| Agree | 2 |  |  |  |
| Disagree | 3 |  |  |  |
| Strongly disagree | 4 |  |  |  |
| Don’t know | 98 |  |  |  |
| Prefer not to say | 99 |  |  |  |
| **411:** The level of violence in this area has increased a lot in the last year | **CODE** |  |  |  |
| Strongly agree | 1 |  |  |  |
| Agree | 2 |  |  |  |
| Disagree | 3 |  |  |  |
| Strongly disagree | 4 |  |  |  |
| Don’t know | 98 |  |  |  |
| Prefer not to say | 99 |  |  |  |
| **412:** The level of safety and security around this facility is similar to where I live. | **CODE** |  |  |  |
| Strongly agree | 1 |  |  |  |
| Agree | 2 |  |  |  |
| Disagree | 3 |  |  |  |
| Strongly disagree | 4 |  |  |  |
| Don’t know | 98 |  |  |  |
| Prefer not to say | 99 |  |  |  |
| ***END OF SECTION 4*** | | | | |

| SECTION 5: CLOSING | |
| --- | --- |
| **501:** Thank you for taking the time to answer my questions. Is there anything else you would like to tell me about your experience giving birth at this facility? | *OPEN TEXT RESPONSE:* |
|  |  |
| As mentioned earlier, any information you have shared will be kept completely confidential. Have a good day! | |
| ***END OF INTERVIEW*** | |

| Data collector comments or observations: |
| --- |
