# Supplementary material for: Understanding women’s care experiences in public health facilities in a conflict-affected area of Nigeria
Source: PLOS Glob Public Health. 2025 Dec 23;5(12):e0004563. doi: 10.1371/journal.pgph.0004563 (PMC12725616; doi:10.1371/journal.pgph.0004563)
Supplement: S2 File — (DOCX) [file pgph.0004563.s002.docx]

**Supplementary File 2: Survey with maternity care providers**

|  |  |
| --- | --- |
| **ASSESSMENT INFO** | |
| Name of data collector: |  |
| Name of team leader: |  |
| Date: (dd/mm/yy) | __ __ / __ __ / __ __ |
| Time: (hh:mm) | __ __ :__ __ |
| **FACILITY TYPE** | |
| Province/State name |  |
| Health zone/LGA name |  |
| City/village name |  |
| Health facility name |  |
| Type of health facility | Tertiary/referral hospital [1]  Secondary/general hospital [2]  Comprehensive health center ................. [3]  Primary health center [4]  Health post [5]  Other (specify)………………………………………..[95]  __________________________________________ |
| Managing authority | Government [1]  Mission/faith based [2]  NGO / Private not-for-profit [3]  Private for profit [4]  Other (specify)………………………………………..[95]  __________________________________________ |

| SECTION 1: PARTICIPANT CHARACTERISTICS | | | | |
| --- | --- | --- | --- | --- |
| **100**: Sex of health worker | **CODE** |  |  |  |
| Male | 1 |  |  |  |
| Female | 2 |  |  |  |
| **101**: Age of health worker | \|  \|  \| \| --- \| --- \| |  |  |  |
| **102**: What is your current occupational category or qualification? | **CODE** |  |  |  |
| Midwife or Nurse midwife | 1 |  |  |  |
| Community midwife | 2 |  |  |  |
| Nurse | 3 |  |  |  |
| Community health extension worker | 4 |  |  |  |
| Community health officer | 5 |  |  |  |
| Clinical officer | 6 |  |  |  |
| Generalist medical doctor | 7 |  |  |  |
| Obstetrician | 8 |  |  |  |
| Pediatrician | 9 |  |  |  |
| Neonatologist  Other specialist medical doctor | 10  11 |  |  |  |
| Student (medical, nursing, midwifery) | 12 |  |  |  |
| Other (specify) | 95 | ______________________________ | | |
| Don’t know | 98 |  |  |  |
| **103**: What year did you graduate (or complete studies) with this qualification?  *(IF NO TECHNICAL QUALIFICATION, ASK “What year did you complete any basic training for your current role in this facility?”* | \|  \|  \|  \|  \| \| --- \| --- \| --- \| --- \| | | | |
| **104**: How many different health facilities have you worked in during the past 3 years? | \|  \|  \| \| --- \| --- \| | | | |
| **105**: What year did you start working in this facility*?* | \|  \|  \|  \|  \| \| --- \| --- \| --- \| --- \| | | | |
| **106**: How long have you worked in your current position*?* | ______ Years  ______ Months | | | |
| **107**: Are you a manager or in-charge for any clinical services? | **CODE** |  |  |  |
| No | 0 |  |  |  |
| Yes | 1 |  |  |  |
| ***END OF SECTION 1.*** | | | | |

| SECTION 2: TRAINING AND SERVICES | | | | | | |
| --- | --- | --- | --- | --- | --- | --- |
| **201**. I would like to ask you a few questions about in-service training you have received related to your work. In-service training refers to on-the-job or continuing professional development training you have received related to your work since you started working. I will start with some general topics*. Note that the training topics I will mention may have been covered as standalone trainings, or they may have been covered under another training topic.*  Have you received any in-service training, training updates, or refresher training in any of the following topics: *(IF YES, ASK: When?)* | **Yes, within past 24 months** | | **Yes, over 24 months ago** | | | **No in-service training or updates** |
| **201a**: Standard precautions, including hand hygiene, cleaning and disinfection, waste management, needle stick and sharp injury prevention | 1 | | 2 | | | 0 |
| **201b**: Training related to injection safety practices or safe injection practices | 1 | | 2 | | | 0 |
| **201c**: Triage and isolation of patients with suspected or confirmed infectious diseases | 1 | | 2 | | | 0 |
| **201d**: Use of personal protective equipment (PPE) to prevent infection at work | 1 | | 2 | | | 0 |
| **201e**: Health Management Information Systems (HMIS) or reporting requirements for maternal and newborn health | 1 | | 2 | | | 0 |
| **201f**: Maternal or perinatal death reviews/audits | 1 | | 2 | | | 0 |
| **201g**: Maternal or perinatal near miss reviews/audits | 1 | | 2 | | | 0 |
| **201h:** Maternity care for women with disabilities | 1 | | 2 | | | 0 |
| **202:** In your current position, and as part of your work for this facility, do you personally provide any antenatal care or postnatal care services**?** | **CODE** |  | |  |  | |
| No | 0 |  | |  | If No 🡪205 | |
| Yes, ANC | 1 |  | |  |  |  |
| Yes, PNC | 2 |  | |  |  |  |
| Yes, both ANC & PNC | 3 |  | |  |  |  |
| **203:**  Have you received any in-service training, training updates or refresher training on topics related to antenatal care? | **CODE** |  | |  |  | |
| No | 0 |  | |  | No 🡪205 | |
| Yes | 1 |  | |  |  | |
| **204.** Have you received any in-service training, training updates, or refresher training in any of the following topics**:** *(IF YES, ASK: When?)* | **Yes, within past 24 months** | | **Yes, over 24 months ago** | | | **No in-service training or updates** |
| **204a**: ANC screening (e.g., blood pressure, urine glucose, and protein) | 1 | | 2 | | | 0 |
| **204b**: Counseling for ANC (e.g., nutrition, FP, and newborn care, early and exclusive breastfeeding) | 1 | | 2 | | | 0 |
| **204c**: Micronutrient supplementation of pregnant women, such as iron and folic acid containing supplements, multiple micronutrient supplements, calcium, and others | 1 | | 2 | | | 0 |
| **204d**: Intermittent preventive treatment of malaria during pregnancy | 1 | | 2 | | | 0 |
| **204e**: Diagnosing and treating sexually transmitted infections (STIs) | 1 | | 2 | | | 0 |
| **204f**: Postpartum family planning counseling | 1 | | 2 | | | 0 |
| **204g**: Gender-based violence screening and response | 1 | | 2 | | | 0 |
| **205:**  Do you personally provide any services to prevent mother-to-child transmission of HIV? | **CODE** |  | |  |  | |
| No | 0 |  | |  |  | |
| Yes | 1 |  | |  |  | |

| 206. Have you received any in-service training, training updates, or refresher training in any of the following topics: *(IF YES, ASK: When?)* | Yes, within past 24 months | | Yes, over 24 months ago | | | No in-service training or updates |
| --- | --- | --- | --- | --- | --- | --- |
| **206a**: HIV testing and counseling | 1 | | 2 | | | 0 |
| **206b**: PMTCT | 1 | | 2 | | | 0 |
| **207:**  In your current position, and as a part of your work for this facility, do you personally provide delivery services? By that I mean assisting in the birth of newborns? | **CODE** |  | |  |  | |
| No | 0 |  | |  |  | |
| Yes | 1 |  | |  |  | |
| **208**: During the past 6 months, approximately how many spontaneous vaginal deliveries have you conducted as the main provider?  *(INCLUDE DELIVERIES AT THIS FACILITY & OUTSIDE/IN PRIVATE PRACTICE)* | \|  \|  \|  \|  \| \| --- \| --- \| --- \| --- \| | | | | | |
| **209**: During the past 6 months, approximately how many assisted vaginal deliveries have you conducted as the main provider?  *(INCLUDE DELIVERIES AT THIS FACILITY & OUTSIDE/IN PRIVATE PRACTICE)* | \|  \|  \|  \|  \| \| --- \| --- \| --- \| --- \| | | | | | |
| **210**: During the past 6 months, approximately how many cesarean deliveries have you conducted as the main provider?  *(INCLUDE DELIVERIES AT THIS FACILITY & ANY OTHER/IN PRIVATE PRACTICE)* | \|  \|  \|  \|  \| \| --- \| --- \| --- \| --- \| | | | | | |
|  | ***If 208 and 209 and 210 = 0 go to Q212*** | | | | | |
| **211:** When was the last time you used a partograph? | **CODE** |  | |  |  | |
| Never | 0 |  | |  |  | |
| Within the past week | 1 |  | |  |  |  |
| Within the past month | 2 |  | |  |  |  |
| Within the past 6 months | 3 |  | |  |  |  |
| Over 6 months ago | 4 |  | |  |  | |
| **212:** How often do you use active management of the third stage of labor (AMTSL) during normal vaginal births? | **CODE** |  | |  |  | |
| Never | 0 |  | |  |  | |
| Rarely | 1 |  | |  |  |  |
| Sometimes | 2 |  | |  |  |  |
| Most of the time | 3 |  | |  |  |  |
| Always | 4 |  | |  |  | |
| **213**: Which of the following procedures are carried out routinely for all patients during labor and delivery at your facility? | **Yes** | **No** | | **Don’t know** |  | |
| **213a:** Artificial rupture of membranes | 1 | 0 | | 98 |  | |
| **213b:** Episiotomy | 1 | 0 | | 98 |  | |
| **213c:** Perineal shaving | 1 | 0 | | 98 |  | |
| **213d:** Maternal blood pressure monitoring | 1 | 0 | | 98 |  | |
| **213e:** Administration of prophylactic antibiotics to women in labor | 1 | 0 | | 98 |  | |
| **213f:** Enema | 1 | 0 | | 98 |  | |
| **213g:** Suction of nose and mouth of newborn | 1 | 0 | | 98 |  | |
| **213h:** Fetal heart rate monitoring | 1 | 0 | | 98 |  | |
| **214:**  Have you received any in-service training, training updates or refresher training on topics related to intrapartum (delivery) care? | **CODE** |  | |  |  | |
| No | 0 |  | |  | If No 🡪216 | |
| Yes | 1 |  | |  |  | |

| 215. Have you received any in-service training, training updates, or refresher training in any of the following topics: Note that the training topics I will mention may have been covered as standalone trainings, or they may have been covered under another training topic.  *(IF YES, ASK: When?)* | Yes, within past 24 months | | Yes, over 24 months ago | | | No in-service training or updates |
| --- | --- | --- | --- | --- | --- | --- |
| **215a**: Integrated Management of Pregnancy and Childbirth (IMPAC) | 1 | | 2 | | | 0 |
| **215b**: Routine care for labor and normal vaginal delivery | 1 | | 2 | | | 0 |
| **215c**: Use of partograph | 1 | | 2 | | | 0 |
| **215d**: Decision making related to premature rupture of membranes (PROM) | 1 | | 2 | | | 0 |
| **215e**: Provision of antenatal corticosteroid for women at risk of preterm birth | 1 | | 2 | | | 0 |
| **215f**: Active management of third stage of labor (AMTSL) | 1 | | 2 | | | 0 |
| **215g**: Basic Emergency obstetric and Newborn care (BEmONC)/ Life saving skills (LSS) | 1 | | 2 | | | 0 |
| **215h**: Comprehensive Emergency Obstetric and Newborn Care (CEmONC) | 1 | | 2 | | | 0 |
| **215i**: Detection and management of pre-eclampsia/eclampsia | 1 | | 2 | | | 0 |
| **215j**: Assisted vaginal delivery (apply vacuum or forceps) | 1 | | 2 | | | 0 |
| **215k**: Manual removal of placenta | 1 | | 2 | | | 0 |
| **215l**: Removal of placenta or products of conception (D&C, vacuum aspiration, etc.) | 1 | | 2 | | | 0 |
| **215m**: Management of postpartum hemorrhage | 1 | | 2 | | | 0 |
| **215n**: Management of puerperal sepsis | 1 | | 2 | | | 0 |
| **215o**: Blood transfusion procedures | 1 | | 2 | | | 0 |
| **215p**: Special delivery care practices for preventing mother-to-child transmission of HIV | 1 | | 2 | | | 0 |
| **215q**: Respectful maternity care (RMC) | 1 | | 2 | | | 0 |
| **216:**  In your current position, and as a part of your work for this facility, do you personally provide care for newborns? | **CODE** |  | |  |  | |
| No | 0 |  | |  |  | |
| Yes | 1 |  | |  |  | |
| **217:**  Have you received any in-service training, training updates or refresher training on topics related to newborn care? | **CODE** |  | |  |  | |
| No | 0 |  | |  | If No --> 301 | |
| Yes | 1 |  | |  |  | |
| **218.** Have you received any in-service training, training updates, or refresher training in any of the following topics: *(IF YES, ASK: When?)* | **Yes, within past 24 months** | | **Yes, over 24 months ago** | | | **No in-service training or updates** |
| **218a**: Essential newborn care | 1 | | 2 | | | 0 |
| **218b**: Early and exclusive breastfeeding | 1 | | 2 | | | 0 |
| **218c**: Neonatal resuscitation using bag and mask | 1 | | 2 | | | 0 |
| **218d**: Kangaroo mother care | 1 | | 2 | | | 0 |
| **218e**: Care for small and sick newborns | 1 | | 2 | | | 0 |
| **218f**: Antibiotics for newborn sepsis | 1 | | 2 | | | 0 |
| **218g**: Nutritional support for newborns (assisted feeding) | 1 | | 2 | | | 0 |
| **218h**: Pain management and palliative care for newborns | 1 | | 2 | | | 0 |
| **218i:**  Counseling and bereavement support for families dealing with stillbirth or newborn death | 1 | | 2 | | | 0 |
| ***END OF SECTION 2.*** | | | | | | |

| SECTION 3: WORKING CONDITIONS | | | | |
| --- | --- | --- | --- | --- |
| **301**: Now I want to ask you a few more questions about your work in this facility. In an average week, how many hours do you work in this facility? | \|  \|  \| \| --- \| --- \| | | | |
| **302:**  The next few questions are about supervision you have personally received. This supervision may have been from a supervisor either from this facility or outside the facility.  Do you receive technical support or supervision in your work related to maternal and newborn care? (*IF YES, ASK*: When was the most recent time?) | **CODE** |  |  |  |
| No | 0 |  |  | If 0 🡪305  If 3 🡪304  If 4 🡪304 |
| Yes, in the last 3 months | 1 |  |  |  |
| Yes, in the last 4-6 months | 2 |  |  |  |
| Yes, in the last 7-12 months | 3 |  |  |  |
| Yes, more than 12 months ago | 4 |  |  |  |
| **303**: How many times in the past 6 months has your work been supervised? | \|  \|  \| \| --- \| --- \| | | | |
| **304**: The last time you were personally supervised, did your supervisor do any of the following: | **Yes** | **No** | **Don’t know** | **Go to** |
| **304a:** Check your reports or record | 1 | 0 | 98 |  |
| **304b:** Observe your work | 1 | 0 | 98 |  |
| **304c:** Provide an opportunity for you to ask questions or request support | 1 | 0 | 98 |  |
| **304d:** Provide any feedback (either positive or negative) on your performance | 1 | 0 | 98 |  |
| **304e:**  Give you verbal or written feedback that you were doing your work well | 1 | 0 | 98 |  |
| **304f:**  Provide updates on administrative or technical issues related to your work | 1 | 0 | 98 |  |
| **304g:**  Discuss problems you have encountered | 1 | 0 | 98 |  |
| **304h:** Discuss clinical skills | 1 | 0 | 98 |  |
| **304i:**  Discuss interpersonal skills | 1 | 0 | 98 |  |
| **305:**  Are there opportunities for promotion in your current job? | **CODE** |  |  |  |
| No | 0 |  |  |  |
| Yes | 1 |  |  |  |
| **306:**  Have you ever been offered any continued professional development opportunities in this facility to facilitate your professional development or move up to career advancement? (*IF YES, ASK*: When was the most recent time?) | **CODE** |  |  |  |
| No | 0 |  |  |  |
| Yes, in the last 6 months | 1 |  |  |  |
| Yes, in the last 7-12 months | 2 |  |  |  |
| Yes, more than 12 months ago | 3 |  |  |  |
| **307**: Do you think that you have equal treatment and opportunities as your colleagues of the opposite sex in terms of: | **Yes** | **No** | **Don’t know** | **Go to** |
| **307a:** Training | 1 | 0 | 98 |  |
| **307b:** Professional advancement | 1 | 0 | 98 |  |
| **307c:** Preferred geographic posts | 1 | 0 | 98 |  |
| **307d:**  Time off | 1 | 0 | 98 |  |
| **307e:**  Work schedule | 1 | 0 | 98 |  |
| **308**: Do you think that you have equal treatment and opportunities as other cadres of health worker in terms of: | **Yes** | **No** | **Don’t know** | **Go to** |
| **30a8:** Training | 1 | 0 | 98 |  |
| **308b:** Professional advancement | 1 | 0 | 98 |  |
| **308c:** Preferred geographic posts | 1 | 0 | 98 |  |
| **308d:**  Time off | 1 | 0 | 98 |  |
| **308e:**  Work schedule | 1 | 0 | 98 |  |
| **309:**  Are you paid salary for the work you do in your current position at this facility? | **CODE** |  |  |  |
| No | 0 |  |  | If No 🡪313 |
| Yes | 1 |  |  |  |
| **309a:**  Is your salary usually paid on time? | **CODE** |  |  |  |
| No | 0 |  |  |  |
| Yes | 1 |  |  |  |
| **310:**  When was the last time you received your salary for the work you do in this facility? | **CODE** |  |  |  |
| Yes, in the last 6 months | 1 |  |  |  |
| Yes, in the last 7-12 months | 2 |  |  |  |
| Yes, more than 12 months ago | 3 |  |  |  |
| **311:**  While working in your current position at this facility, have you received any salary top-up or supplementary payment for the work you do? (*IF YES, PROBE*: When was the last time you received a monetary salary supplement?) | **CODE** |  |  |  |
| No | 0 |  |  | If No 🡪313 |
| Yes, in the last 6 months | 1 |  |  |  |
| Yes, in the last 7-12 months | 2 |  |  |  |
| Yes, more than 12 months ago | 3 |  |  |  |
| **312:**  Which types of salary top-ups or supplementary payment did you receive? | **CODE** |  |  |  |
| Monthly or daily salary top-up | 1 |  |  |  |
| Perdiem when attending training | 2 |  |  |  |
| Daily allowance | 3 |  |  |  |
| Other (specifcy) | 95_______________________________ | | | |
| **313:**  While working in your current position at this facility, have you received any non-monetary compensation or incentives for the work you do? (*IF YES, PROBE*: When was the most recent time?) | **CODE** |  |  |  |
| No | 0 |  |  | If No 🡪315 |
| Yes, in the last 6 months | 1 |  |  |  |
| Yes, in the last 7-12 months | 2 |  |  |  |
| Yes, more than 12 months ago | 3 |  |  |  |
| **314:**  What non-monetary incentives have you received? | **CODE**  **NO** | **YES** |  |  |
| Time off/vacation | 0 | 1 |  |  |
| Uniforms, backpacks, caps, etc | 0 | 1 |  |  |
| Discounted medicines, free care for you or your family, vouchers, etc | 0 | 1 |  |  |
| Training | 0 | 1 |  |  |
| Food rations | 0 | 1 |  |  |
| Subsidized housing  None of the above | 0  0 | 1  1 |  |  |
| Other (specify) | 0 1 | | | ______________ |
| **315:**  Now I would like to talk about your time working in this facility. Would you say that you are very satisfied, satisfied, neither satisfied or dissatisfied, not satisfied, or very dissatisfied about working here? | **CODE** |  |  |  |
| Very satisfied | 1 |  |  |  |
| Satisfied | 2 |  |  |  |
| Neither satisfied nor dissatisfied | 3 |  |  |  |
| Not satisfied | 4 |  |  |  |
| Very dissatisfied | 5 |  |  |  |
| Declined to answer | 99 |  |  |  |

| 316: Among the various things related to your working situation that you would like to see improved, which three things would most improve your ability to provide good quality of care services? Please rank them in order of importance, with 1 being the most important.  *(DO NOT READ CHOICES TO YOUR RESPONDENT)* | | Choice 1 | | | ___________________ | | |  |
| --- | --- | --- | --- | --- | --- | --- | --- | --- |
|  |  | **Choice 2** | | | **___________________** | | |  |
|  |  | **Choice 3** | | | **___________________** | | |  |
| a. More support from supervisor  b. More trainings / updates  c. More supplies / stocks  d. Better quality equipment / supplies  e. Less workload / more staff  f. Better working hours / flexible times  g. More incentives (financial or other) | h. Transportation for referral patients  i. Increased security  j. Better facility infrastructure  k. More autonomy/independence  l. Support/counseling for staff  m. Social activities for staff  o. Other |  |  |  |  |  |  |  |
| ***READ TO THE RESPONDENT*:** Now I would like to ask you some additional questions about working at this facility. You may find some of these questions very personal. However, your answers are crucial for helping to understand the experiences of health workers in this area. Let me assure you that your answers are completely confidential and will not be told to anyone and no one else in your health facility will know that you were asked these questions. If I ask you any question you don't want to answer, just let me know and I will go on to the next question.  **317:**  Research has shown that maternity care providers are sometimes treated badly or experience poor conditions at work. Do you think disrespect between health workers or cadres of health workers is an issue in this facility? | | **CODE** |  |  | |  | |  |
| Disrespect is not an issue. Health workers treat each other very respectfully | | 1 |  |  | |  | |  |
| Disrespect is sometimes an issue. Some health workers are treated disrespectfully. | | 2 |  |  | |  |  |  |
| Disrespect is an issue. Health workers treat each other disrespectfully. | | 3 |  |  | |  |  |  |
| Don’t know | | 98 |  |  | |  | |  |
| Declined to answer | | 99 |  |  | |  |  |  |
| **318**: At any time during your work in this facility, did any other staff member: | | **Yes** | **No** | **Decline to answer** | | |  |  |
| **318a:** Slap you | | 1 | 0 | 99 | | |  |  |
| **318b:** Hit or punch you | | 1 | 0 | 99 | | |  |  |
| **318c:** Physically threaten you | | 1 | 0 | 99 | | |  |  |
| **318d:** Physically mistreat or harm you | | 1 | 0 | 99 | | |  |  |
| **318e:** Shout at you | | 1 | 0 | 99 | | |  |  |
| **318f:** Say or do something to humiliate or harass you | | 1 | 0 | 99 | | |  |  |
| **318g:** Verbally threaten you | | 1 | 0 | 99 | | |  |  |
| **318h:** Verbally mistreat or psychologically abuse you in any other way | | 1 | 0 | 99 | | |  |  |
| **318j:** Force or pressure you to perform sexual acts | | 1 | 0 | 99 | | |  |  |
| **318k:** Accuse you of acting in bad faith or intentionally causing harm | | 1 | 0 | 99 | | |  |  |
| **319**: At any time during your work in this facility, did any patient or their family: | | **Yes** | **No** | **Decline to answer** | | |  |  |
| **319a:** Slap you | | 1 | 0 | 99 | | |  |  |
| **319b:** Hit or punch you | | 1 | 0 | 99 | | |  |  |
| **319c:** Physically threaten you | | 1 | 0 | 99 | | |  |  |
| **319d:** Physically mistreat or harm you | | 1 | 0 | 99 | | |  |  |
| **319e:** Shout at you | | 1 | 0 | 99 | | |  |  |
| **319f:** Say or do something to humiliate or harrass you | | 1 | 0 | 99 | | |  |  |
| **319g:** Verbally threaten you | | 1 | 0 | 99 | | |  |  |
| **319h:** Verbally mistreat or psychologically abuse you in any other way | | 1 | 0 | 99 | | |  |  |
| **319j:** Force or pressure you to perform sexual acts | | 1 | 0 | 99 | | |  |  |
| **318k:** Accuse you of acting in bad faith or intentionally causing harm | | 1 | 0 | 99 | | |  |  |
| ***END OF SECTION 3.*** | | | | | | | |  |
| SECTION 4: MATERNAL AND NEWBORN HEALTH KNOWLEDGE | | | | | | | | |
| *FOR THE FOLLOWING QUESTIONS, READ THE QUESTION ALOUD TO THE HEALTH WORKER. DO NOT READ THE ANSWER CHOICES ALOUD. IF YOU ARE NOT SURE WHETHER AN ANSWER GIVEN BY HEALTH WORKER MATCHES A LISTED ANSWER, PROBE FOR MORE DETAIL. IF THEY GIVE AN ANSWER THAT IS NOT LISTED, MOVE ON TO THEIR NEXT ANSWER. USE THE PROBE TO ENCOURAGE HEALTH WORKER TO* ***GIVE AS MANY ANSWERS AS THEY CAN THINK OF****.* | | | | | | | | |
| ***READ TO THE RESPONDENT***: Now I would like to ask some questions about your personal knowledge of maternal and newborn care guidelines and procedures. Please answer the following questions to the best of your knowledge according to your training, even if some of your responses are not part of your routine practice at this facility. Most of the questions I ask you will require multiple responses identifying multiple steps or interventions. Assume all needed supplies, medications, and equipment are available. When thinking about your answers, you should include actions or interventions that should be performed at your facility or a referral facility, even if they don’t always happen. I will probe sometimes to help you remember some more information. Please provide all responses that come to mind. | | | | | | | | |
| **401**: What the key steps for active management of the third stage of labor (AMTSL)? *(SELECT ALL MENTIONED. PROBE. DO NOT READ OPTIONS)* | | Mentioned? | | | | | | |
|  |  | **Yes** | **No** |  | |  | | |
| **401a:**  Administer a uterotonic immediately/ within 1 min of birth | | 1 | 0 |  | |  | | |
| **401b:**  Administer a uterotonic with delivery of anterior shoulder | | 1 | 0 |  | |  | | |
| **401c:**  Administration of a uterotonic after delivery of placenta and checking for second baby | | 1 | 0 |  | |  | | |
| **401d:**  Controlled cord traction | | 1 | 0 |  | |  | | |
| **401e:**  Check uterine tone and massage if soft  **401f:** Other (specify) | | 1  1 | 0  0 | ______ | | _____________ | | |
| **402**: What actions during labor and delivery would you take in an HIV+ woman to prevent or reduce mother-to-child transmission of the virus? *(SELECT ALL MENTIONED. PROBE IF ANYTHING ELSE. DO NOT READ OPTIONS)* | | **Yes** | **No** |  | |  | | |
| **402a:** PMTCT counseling | | 1 | 0 |  | |  | | |
| **402b:**  Provide ARV prophylaxis to women in early labor | | 1 | 0 |  | |  | | |
| **402c:**  No routine episiotomy | | 1 | 0 |  | |  | | |
| **402d:**  Minimize instrument delivery | | 1 | 0 |  | |  | | |
| **402e:**  Hibitane vaginal cleansing | | 1 | 0 |  | |  | | |
| **402f:** Minimize vaginal exam | | 1 | 0 |  | |  | | |
| **402g:**  Minimize artificial rupture of membranes | | 1 | 0 |  | |  | | |
| **402h:**  Appropriate use of partograph | | 1 | 0 |  | |  | | |
| **402i:**  Active management of third stage of labor | | 1 | 0 |  | |  | | |
| **402j:**  Avoid milking cord/immediate clamp cord | | 1 | 0 |  | |  | | |
| **402k:**  Provide ARV prophylaxis to infant | | 1 | 0 |  | |  | | |
| **402l:**  Other (specify) | | 1 | 0 ________________________ | | | | | |
| **403:** When a baby is delivered and there is no complication, what care is important to give them immediately after birth and in the first hour? *(SELECT ALL MENTIONED. PROBE IF ANYTHING ELSE. DO NOT READ OPTIONS)* | |  | |  | |  | | |
|  |  | **Yes** | **No** |  | |  | | |
| **403a:** Wipe face after birth of head | | 1 | 0 |  | |  | | |
| **403b:**  Ensure baby was breathing / crying | | 1 | 0 |  | |  | | |
| **403c:**  Provide thermal protection (skin to skin) | | 1 | 0 |  | |  | | |
| **403d:**  Bathe newborn shortly after birth | | 1 | 0 |  | |  | | |
| **403e:**  Suction newborn with bulb | | 1 | 0 |  | |  | | |
| **403f:** Ensure mother initiates breastfeeding | | 1 | 0 |  | |  | | |
| **403g:**  Assess/examine newborn within 1 hour | | 1 | 0 |  | |  | | |
| **403h:**  Weigh newborn | | 1 | 0 |  | |  | | |
| **403i:**  Provide eye prophylaxis / antibiotic ointment | | 1 | 0 |  | |  | | |
| **403j:**  Provide prelacteal feed / water | | 1 | 0 |  | |  | | |
| **403k:**  Cut cord with sterile blade / scissors | | 1 | 0 |  | |  | | |
| **403l:**  Apply antiseptic or other material to cord stump | | 1 | 0 |  | |  | | |
| **403m:**  Vitamin K injection | | 1 | 0 |  | |  | | |
| **403n:**  Other (specify) | | 1 | 0 ________________________ | | | | | |
| 404: What are the clinical signs and symptoms of birth asphyxia? *(SELECT ALL MENTIONED. PROBE IF ANYTHING ELSE. DO NOT READ OPTIONS)* | | Mentioned? | |  | |  | | |
|  |  | **Yes** | **No** |  | |  | | |
| **404a:** Depressed breathing | | 1 | 0 |  | |  | | |
| **404b:**  Floppiness | | 1 | 0 |  | |  | | |
| **404c:**  Heart rate below 100 beats per minute | | 1 | 0 |  | |  | | |
| **404d:**  Central cyanosis (blue tongue)  **404e:** Other (specify) | | 1  1 | 0  0 | ________ | | ______________ | | |
| **405:** What are the steps to resuscitate a baby who is not breathing, if rubbing the back does not help? *(SELECT ALL MENTIONED. PROBE IF ANYTHING ELSE. DO NOT READ OPTIONS)* | |  | |  | |  | | |
|  |  | **Yes** | **No** |  | |  | | |
| **405a:** Call for help | | 1 | 0 |  | |  | | |
| **405b:** Explain to mother the condition of baby | | 1 | 0 |  | |  | | |
| **405c:** Place newborn face up | | 1 | 0 |  | |  | | |
| **405d:** Wrap or cover baby, except for face and upper portion of chest | | 1 | 0 |  | |  | | |
| **405e:**  Position baby’s head so neck is slightly extended | | 1 | 0 |  | |  | | |
| **405f:** Suction nose and mouth is airway blocked | | 1 | 0 |  | |  | | |
| **405g:** Start ventilation using bag and mask  **405h:** Other (specify) | | 1  1 | 0  0 | _______ | | ___________ | | |
| **406**: What do you check for when assessing a baby prior to discharge? *(SELECT ALL MENTIONED. PROBE IF ANYTHING ELSE. DO NOT READ OPTIONS)* | |  | |  | |  | | |
|  |  | **Yes** | **No** |  | |  | | |
| **406a**: Baby breastfeeding well | | 1 | 0 |  | |  | | |
| **406b**: Proper positioning for breastfeeding | | 1 | 0 |  | |  | | |
| **406c:** Color tone of baby | | 1 | 0 |  | |  | | |
| **406d:**  Signs of infection (fever) | | 1 | 0 |  | |  | | |
| **406e:**  Difficulty breathing | | 1 | 0 |  | |  | | |
| **406f:** Eye swelling or discharge | | 1 | 0 |  | |  | | |
| **406g:**  Umbilical cord | | 1 | 0 |  | |  | | |
| **406h:**  Baby’s weight | | 1 | 0 |  | |  | | |
| **406i:** Alertness of baby  **406j:** Other (specify) | | 1  1 | 0  0 |  | | ______________ | | |
| **407**: What do you check for when assessing a mother prior to discharge? *(SELECT ALL MENTIONED. PROBE IF ANYTHING ELSE. DO NOT READ OPTIONS)* | |  | |  | |  | | |
|  |  | **Yes** | **No** |  | |  | | |
| **407a:** Vaginal bleeding | | 1 | 0 |  | |  | | |
| **407b:**  Signs of infection (fever) | | 1 | 0 |  | |  | | |
| **407c:**  Blood pressure | | 1 | 0 |  | |  | | |
| **407d:**  Abdominal tenderness | | 1 | 0 |  | |  | | |
| **407e:**  Size and firmness of uterus | | 1 | 0 |  | |  | | |
| **407f:** Drop vein thrombosis | | 1 | 0 |  | |  | | |
| **407g:**  Breast engorgement | | 1 | 0 |  | |  | | |
| **407h:**  Signs of anemia | | 1 | 0 |  | |  | | |
| **407i:**  Lochia (vaginal discharge) | | 1 | 0 |  | |  | | |
| **407j:**  Signs of depression | | 1 | 0 |  | |  | | |
| **407k:** Dribbling urine | | 1 | 0 |  | |  | | |
| **407l:**  Cough or breathing difficulties | | 1 | 0 |  | |  | | |
| **407m:**  Signs of malnutrition or food insecurity  **407n:** Other (specify) | | 1  1 | 0  0 |  | | ______________ | | |
| **408:** What actions are appropriate for a woman who presents with, or develops heavy bleeding postpartum from atonic/uncontracted uterus? *(SELECT ALL MENTIONED. PROBE IF ANYTHING ELSE. DO NOT READ OPTIONS)* | | **Yes** | **No** |  | |  | | |
| **408a:** Massage the fundus | | 1 | 0 |  | |  | | |
| **408b:**  Empty urinary bladder | | 1 | 0 |  | |  | | |
| **408c:**  Give uterotonics, IM or IV | | 1 | 0 |  | |  | | |
| **408d:**  Give misoprostol, oral or rectal | | 1 | 0 |  | |  | | |
| **408e:**  Give tranexamic acid | | 1 | 0 |  | |  | | |
| **408f:** Perform bimannual compression of uterus | | 1 | 0 |  | |  | | |
| **408g:**  Perform abdominal compression of aorta | | 1 | 0 |  | |  | | |
| **408h:**  Start IV fluids | | 1 | 0 |  | |  | | |
| **408i:**  Take blood for hemoglobin grouping and x-matching | | 1 | 0 |  | |  | | |
| **408j:**  Raise foot of bed | | 1 | 0 |  | |  | | |
| **408k:**  Insert uterine balloon tamponade | | 1 | 0 |  | |  | | |
| **408l:** Refer to doctor or hospital | | 1 | 0 |  | |  | | |
| **408m:**  Other (specify) | | 1 | 0 ___________________ | | | | | |
| **409:** When should membranes be ruptured artificially by the provider? *(SELECT ALL MENTIONED. PROBE IF ANYTHING ELSE. DO NOT READ OPTIONS)* | | **Yes** | **No** |  | |  | | |
| **409a:** At start of second stage | | 1 | 0 |  | |  | | |
| **409b:**  Immediately prior to delivery when they are bulging in vagina | | 1 | 0 |  | |  | | |
| **409c:**  Routinely during active phase of labor | | 1 | 0 |  | |  | | |
| **409d:**  As part of augmentation of labor | | 1 | 0 |  | |  | | |
| **409e:**  Upon admission for all women | | 1 | 0 |  | |  | | |
| **409f:** To check color of fluid/liquor when fetal distress is noted | | 1 | 0 |  | |  | | |
| **409g:** Not to be ruptured | | 1 | 0 |  | |  | | |
| **410**: What actions do you believe are most appropriate in managing a woman with severe pre-eclampsia at term? *(SELECT ALL MENTIONED. PROBE IF ANYTHING ELSE. DO NOT READ OPTIONS)* | | Yes | No |  | |  | | |
| **410a:** Provide magnesium sulphate | | 1 | 0 |  | |  | | |
| **410b:**  Provide diazepam | | 1 | 0 |  | |  | | |
| **410c:**  Provide anti-hypertensives | | 1 | 0 |  | |  | | |
| **410d:**  Prepare to deliver within 24 hours  **410e:** Other (specify) | | 1  1 | 0  0 |  | | ______________ | | |
| **411:** Which antibiotics would you give to a woman who is diagnosed by postpartum endometritis following a vaginal delivery? *(SELECT ALL MENTIONED. PROBE IF ANYTHING ELSE. DO NOT READ OPTIONS)* | | **Yes** | **No** |  | |  | | |
| **411a:** Ampicillin | | 1 | 0 |  | |  | | |
| **411b:** Gentamicin | | 1 | 0 |  | |  | | |
| **411c:**  Metronidazole | | 1 | 0 |  | |  | | |
| **411d:**  Other antibiotic (specify) | | 1 | 0 |  | | ______________ | | |
| **412:** When you see a woman with complications from incomplete abortion (needing post-abortion care), what do you do? *(SELECT ALL MENTIONED. PROBE IF ANYTHING ELSE. DO NOT READ OPTIONS)* | | **Yes** | **No** |  | |  | | |
| **412a:** Do a vaginal exam | | 1 | 0 |  | |  | | |
| **412b:** Assess vaginal bleeding | | 1 | 0 |  | |  | | |
| **412c:**  Assess vital signs | | 1 | 0 |  | |  | | |
| **412d:**  Begin IV fluids | | 1 | 0 |  | |  | | |
| **412e:** Begin antibiotics | | 1 | 0 |  | |  | | |
| **412f:** Treat with uterotonics | | 1 | 0 |  | |  | | |
| **412g:** Manual/digital removal POC | | 1 | 0 |  | |  | | |
| **412f:** Perform (manual/electric) vacuum aspiration | | 1 | 0 |  | |  | | |
| **412g:**  Perform dilation with curettage or evacuation | | 1 | 0 |  | |  | | |
| **412h:**  Provide counseling | | 1 | 0 |  | |  | | |
| **412i:** Refer | | 1 | 0 |  | |  | | |
| **412j:** Other (specify) | | 1 | 0 ___________________ | | | | | |
| **413:** What counseling or information do you give patients who were treated for an incomplete abortion? *(SELECT ALL MENTIONED. PROBE IF ANYTHING ELSE. DO NOT READ OPTIONS)* | | Yes | No |  | |  | | |
| **413a:** Information on how to prevent reproductive tract infection/HIV | | 1 | 0 |  | |  | | |
| **413b:** Information on when a woman can conceive again | | 1 | 0 |  | |  | | |
| **413c:**  Counseling on family planning methods | | 1 | 0 |  | |  | | |
| **413d:**  Information on social support | | 1 | 0 |  | |  | | |
| **413e:**  Information about the consequences of an unsafe abortion | | 1 | 0 |  | |  | | |
| **413f:**  Information about safe abortion and how to obtain one in the future | | 1 | 0 |  | |  | | |
| **413j:** Other (specify) | | 1 | 0 _________________ | | | | | |
| **414:** When a woman presents as a recent survivor of rape, what do you do? *(SELECT ALL MENTIONED. PROBE IF ANYTHING ELSE. DO NOT READ OPTIONS)* | | Yes | No |  | |  | | |
| **414a:**  HIV testing and counseling | | 1 | 0 |  | |  | | |
| **414b:** Vaginal smear/swab | | 1 | 0 |  | |  | | |
| **414c:**  Provide emergency contraception | | 1 | 0 |  | |  | | |
| **414d:**  Provide post-exposure prophylaxis for HIV | | 1 | 0 |  | |  | | |
| **414e:**  Refer for mental health and psychosocial support | | 1 | 0 |  | |  | | |
| **414f:** Refer for social support services | | 1 | 0 |  | |  | | |
| **414f:** Other (specify) | | 1 | 0 ______________________ | | | | | |
| ***READ TO THE RESPONDENT:*** *Now I would like to present you with a scenario you might encounter on the job. A woman is brought to the emergency department of the district hospital by her husband after she complained of a severe headache and blurred vision. She is 20 years old, this is her first pregnancy, and she is 37 weeks gestation. She had 2 ANC visits and no problems. Fetal movement is normal. Her bp is 160/120. She has contractions at a rate of 2 in 10 minutes, lasting 20 seconds by palpation. Her urine has 3+ protein.*  **415**: Given the information presented above, what is your working diagnosis? (*DO NOT READ ANSWERS ALOUD AND CHOOSE ONLY 1 ANSWER)* | | **CODE** |  |  | |  | | |
| **415a:** Kidney infection | | 1 |  |  | |  | | |
| **415b:** Severe pre-eclampsia | | 2 |  |  | |  | | |
| **415c:**  Malaria | | 3 |  |  | |  | | |
| **415d:**  Eclampsia | | 4 |  |  | |  | | |
| **415e:**  In labor | | 5 |  |  | |  | | |
| **415f:**  Other (specify) | | 95 |  | | | | | |
| **419g:**  Don’t know | | 98 |  |  | |  | | |
| **416**: When a newborn weighs less than 2500 grams, what special care do you provide? *(SELECT ALL MENTIONED. PROBE IF ANYTHING ELSE. DO NOT READ OPTIONS)* | |  | |  | |  | | |
|  |  | **Yes** | **No** |  | |  | | |
| **416a:** Ensure that the baby is warm with skin-to-skin with mother (kangaroo technique) | | 1 | 0 |  | |  | | |
| **416b:**  Ensure baby is warm by placing baby in incubator/radiant warmer | | 1 | 0 |  | |  | | |
| **416c:**  Provide extra support to establish breastfeeding (positioning, etc) | | 1 | 0 |  | |  | | |
| **416d:**  Monitor ability to breastfeed | | 1 | 0 |  | |  | | |
| **416e:** Assess for jaundice | | 1 | 0 |  | |  | | |
| **416f:** Assess for breathing difficulties (need for oxygen supplementation) | | 1 | 0 |  | |  | | |
| **416g:**  Monitor baby for the first 24 hours | | 1 | 0 |  | |  | | |
| **416h:**  Ensure infection prevention | | 1 | 0 |  | |  | | |
| **416i:** Other (specify) | | 1 | 0 _______________ | | | | | |
| **417**: What are the signs and symptoms of severe infection in a newborn? *(SELECT ALL MENTIONED. PROBE IF ANYTHING ELSE. DO NOT READ OPTIONS)* | |  | |  | |  | | |
|  |  | **Yes** | **No** |  | |  | | |
| **417a:** Poor/no breathing | | 1 | 0 |  | |  | | |
| **417b:**  Restlessness / irritability | | 1 | 0 |  | |  | | |
| **417c:**  Hypothermia | | 1 | 0 |  | |  | | |
| **417d:**  Hyperthermia | | 1 | 0 |  | |  | | |
| **417e:**  Breathing rating >60/minute | | 1 | 0 |  | |  | | |
| **417f:** Convulsions | | 1 | 0 |  | |  | | |
| **417g:** Puss/redness around umbilicus | | 1 | 0 |  | |  | | |
| **417g:**  Abcess on any part of body | | 1 | 0 |  | |  | | |
| **417h:**  Skin pustules | | 1 | 0 |  | |  | | |
| **417i:**  Lethargy / no movement (conscious) | | 1 | 0 |  | |  | | |
| **417j:** Poor/no feeding | | 1 | 0 |  | |  | | |
| **417k:** Poor /no urine output | | 1 | 0 |  | |  | | |
| **417l:** Other (specify) | | 1 | 0 ______________________ | | | | | |
| **418**: What actions should be taken to care for a woman who has just had a stillbirth or newborn death? *(SELECT ALL MENTIONED. PROBE IF ANYTHING ELSE. DO NOT READ OPTIONS)* | |  | |  | |  | | |
|  |  | **Yes** | **No** |  | |  | | |
| **418a:** Ensure privacy | | 1 | 0 |  | |  | | |
| **418b:** Supportive listening | | 1 | 0 |  | |  | | |
| **418c:** Honest, respectful and culturally appropriate communication | | 1 | 0 |  | |  | | |
| **418d:**  Be available for questions from the woman and her family | | 1 | 0 |  | |  | | |
| **418e:**  Support to understand decisions that need to be made related to the death (including taking the baby home or not, making funeral arrangements, etc) | | 1 | 0 |  | |  | | |
| **418f:** Investigating/identifying causes and factors contributing to death | | 1 | 0 |  | |  | | |
| **418g:** Acknowledge the death and variety of the client’s grief response (without minimizing the loss) | | 1 | 0 |  | |  | | |
| **418h:**  Offer information and postnatal care to address physical and psychological needs | | 1 | 0 |  | |  | | |
| **418i:** Refer to social worker or bereavement support team for continued care | | 1 | 0 |  | |  | | |
| **418j:**  Follow up phone call after discharge | | 1 | 0 |  | |  | | |
| **418k:** Provide information about future pregnancy | | 1 | 0 |  | |  | | |
| **418l:** Other (specify) | | 1 | 0 ______________________ | | | | | |
| ***READ TO THE RESPONDENT***: *Now I am going to read you a few statements. For each one, please tell me if you strongly agree, agree, disagree, or strongly disagree.*  **419:** My routine practice includes the best practices I have described in the previous questions. | |  | |  | |  | | |
|  |  | **CODE** |  |  | |  | | |
| Strongly agree | | 1 |  |  | |  | | |
| Agree | | 2 |  |  | |  | | |
| Disagree | | 3 |  |  | |  | | |
| Strongly disagree | | 4 |  |  | |  | | |
| Don’t know | | 98 |  |  | |  | | |
| Prefer not to say | | 99 |  |  | |  | | |
| **420:** Most staff at this facility demonstrate knowledge of best practices and routinely implement those practices in their clinical care. | |  | |  | |  | | |
|  |  | **CODE** |  |  | |  | | |
| Strongly agree | | 1 |  |  | |  | | |
| Agree | | 2 |  |  | |  | | |
| Disagree | | 3 |  |  | |  | | |
| Strongly disagree | | 4 |  |  | |  | | |
| Don’t know | | 98 |  |  | |  | | |
| Prefer not to say | | 99 |  |  | |  | | |
| ***END OF SECTION 4*** | | | | | | | | |

| SECTION 5: PERSONAL EXPERIENCES & PERSPECTIVES | | | | | |
| --- | --- | --- | --- | --- | --- |
| **501:**  Treating clients with respect and dignity during labor, birth and after birth is a health worker’s duty. In your own words, how do you define respectful and dignified maternity care? | **Response:** | | | | |
| **502:** To what extent does the environment here allow you to provide respectful maternity care? | **Response:** | | | | |
|  |  |  |  |  | |
| **502a:** What aspects of the environment limit your ability to provide respectful maternity care? | **Response:** | | | | |
| **503:**  Overall, do you feel well-prepared and trained for your current position? | **CODE** |  |  |  | |
| No | 0 |  |  |  | |
| Yes | 1 |  |  |  |  |
| Somewhat | 2 |  |  |  |  |
| **504:**  Do you ever have to perform tasks that you were not trained for? | **CODE** |  |  |  | |
| No, Never | 0 |  | If No or no answer 🡪506 | | |
| Yes, often | 1 |  |  |  | |
| Yes, sometimes | 2 |  |  |  | |
| Prefer not to answer | 99 |  |  |  | |
| **505:** Can you provide an example of tasks you are performing that you were not trained for? | **Response:** | | | | |
| **506:** How has insecurity affected the provision of health services in this facility? | **Response:** | | | | |
| ***READ TO RESPONDENT:*** I just have a few final questions for you. I am going to read you a sentence. Please tell me if you strongly agree, agree, disagree, or strongly disagree with each statement.  **507:** I feel safe from crime and violence when I am at the facility. | **CODE** |  |  | |  |
| Strongly agree | 1 |  |  | |  |
| Agree | 2 |  |  | |  |
| Disagree | 3 |  |  | |  |
| Strongly disagree | 4 |  |  | |  |
| Don’t know | 98 |  |  | |  |
| Prefer not to say | 99 |  |  | |  |
| **508:** I feel safe from crime and violence when traveling to and from work at the facility. | **CODE** |  |  | |  |
| Strongly agree | 1 |  |  | |  |
| Agree | 2 |  |  | |  |
| Disagree | 3 |  |  | |  |
| Strongly disagree | 4 |  |  | |  |
| Don’t know | 98 |  |  | |  |
| Prefer not to say | 99 |  |  | |  |
| **509:** The area surrounding this health facility is marked by repeated violence. | **CODE** |  |  | |  |
| Strongly agree | 1 |  |  | |  |
| Agree | 2 |  |  | |  |
| Disagree | 3 |  |  | |  |
| Strongly disagree | 4 |  |  | |  |
| Don’t know | 98 |  |  | |  |
| Prefer not to say | 99 |  |  | |  |
| **510**: The level of violence in this area has increased a lot in the last year. | CODE |  |  | |  |
| Strongly agree | 1 |  |  | |  |
| Agree | 2 |  |  | |  |
| Disagree | 3 |  |  | |  |
| Strongly disagree | 4 |  |  | |  |
| Don’t know | 98 |  |  | |  |
| Prefer not to say | 99 |  |  | |  |
| **511:** The level of safety and security around this facility is similar to where I live. | **CODE** |  |  | |  |
| Strongly agree | 1 |  |  | |  |
| Agree | 2 |  |  | |  |
| Disagree | 3 |  |  | |  |
| Strongly disagree | 4 |  |  | |  |
| Don’t know | 98 |  |  | |  |
| Prefer not to say | 99 |  |  | |  |
| **512:** I am respected by colleagues at this facility. | **CODE** |  |  | |  |
| Strongly agree | 1 |  |  | |  |
| Agree | 2 |  |  | |  |
| Disagree | 3 |  |  | |  |
| Strongly disagree | 4 |  |  | |  |
| Don’t know | 98 |  |  | |  |
| Prefer not to say | 99 |  |  | |  |
| **513:** I am respected in the community. | **CODE** |  |  | |  |
| Strongly agree | 1 |  |  | |  |
| Agree | 2 |  |  | |  |
| Disagree | 3 |  |  | |  |
| Strongly disagree | 4 |  |  | |  |
| Don’t know | 98 |  |  | |  |
| Prefer not to say | 99 |  |  | |  |
| **514:**  I am fulfilling my full competence as a maternal and newborn heath provider. | **CODE** |  |  | |  |
| Strongly agree | 1 |  |  | |  |
| Agree | 2 |  |  | |  |
| Disagree | 3 |  |  | |  |
| Strongly disagree | 4 |  |  | |  |
| Don’t know | 98 |  |  | |  |
| Prefer not to say | 99 |  |  | |  |
| ***END OF SECTION 5. END OF INTERVIEW.*** | | | | | |
| ***Data collector comments and observations: Please note any challenges in completing tool (if interrupted, staff not cooperative, etc) or problems with specific questions.*** | | | | | |
| ***END OF TOOL B*** | | | | | |
